# *Fmr1*-KO mice failure to detect object novelty associates with a post-test decrease of structural and synaptic plasticity upstream of the hippocampus

**DOI:** 10.1038/s41598-023-27991-9

**Published:** 2023-01-14

**Authors:** Antonella Borreca, Mariassunta De Luca, Antonella Ferrante, Zaira Boussadia, Annabella Pignataro, Alberto Martire, Martine Ammassari-Teule

**Affiliations:** 1 Institute of Neuroscience, CNR-National Research Council, Vedano Al Lambro, Milan, Italy; 2 Humanitas Clinical and Research Center-IRCCS, Via Manzoni 56, Rozzano, 20090 Milan, Italy; 3 European Center for Brain Research, FSL-Santa Lucia Foundation, Rome, Italy; 4 National Center for Drug Research and Evaluation, ISS-Istituto Superiore di Sanità, Rome, Italy; 5 Institute of Translational Pharmacology, CNR-National Research Council, Rome, Italy; 6 Institute of Biochemistry and Cell Biology, CNR-National Research Council, Monterotondo, Rome, Italy

**Keywords:** Cognitive neuroscience, Diseases of the nervous system

## Abstract

Mice with deletion of the *FMR1* gene show episodic memory impairments and exhibit dendritic spines and synaptic plasticity defects prevalently identified in non-training conditions. Based on evidence that synaptic changes associated with normal or abnormal memory emerge when mice are cognitively challenged, here we examine whether, and how, fragile entorhinal and hippocampal synapses are remodeled when mice succeed or fail to learn. We trained *Fmr1* knockout (KO) and wild-type C57BL/6J (WT) mice in the novel object recognition (NOR) paradigm with 1 h or 24 h training-to-test intervals and then assessed whether varying the time between the presentation of similar and different objects modulates NOR performance and plasticity along the entorhinal cortex-hippocampus axis. At the 1 h-interval, KO mice failed to discriminate the novel object, showed a collapse of spines in the lateral entorhinal cortex (LEC), and of long-term potentiation (LTP) in the lateral perforant path (LPP), but a normal increase in hippocampal spines. At the 24 h, they exhibited intact NOR performance, typical LEC and hippocampal spines, and exaggerated LPP-LTP. Our findings reveal that the inability of mice to detect object novelty primarily stands in their impediment to elaborate, and convey to the hippocampus, sensory/perceptive object representations.

## Introduction

Fragile X syndrome (FXS) is a neurodevelopmental disorder due to mutations in the Fragile X messenger ribonucleoprotein 1 (*FMR1*) gene which prevent transcription of the Fragile X Messenger Ribonucleoprotein (FMRP). A major neuropathological feature of FXS is the presence of abundant immature dendritic spines in cortical and subcortical regions^1–3^ which alters the cytoarchitecture and the wiring diagram of neural circuits implicated in sensory, motor, emotional, and cognitive functions. These alterations are at the origin of behavioral disturbances ranging from atypical sensory processing, inattention, impulsivity, repetitive movements, and cognitive inflexibility, which culminate in the manifestation of social and intellectual disabilities^4^. Mice with deletion of the *FMR1* gene (KO mice) also show greater spine density with a majority of immature-appearing spines in cortical and subcortical regions critical for sensory processing and episodic memory^5–11^. Consistently, they exhibit episodic memory impairments^12–19^ even though observed in the massed training regimen, which suggests that their memory deficit depends more on a delay in learning than on incapability to learn^20–23^. In healthy conditions, acquisition and storage of information require induction and stabilization of synaptic changes in memory-encoding regions supported by an augmentation in the number and size of dendritic spines^24–27^ and an increment of synaptic strength^28,29^. A close link exists between the structure of spines and synaptic activity^30^. Small spines host weak synapses^31^ and the immature spines of KO mice underlie synaptic dysfunctions. Among those, impairments in basal synaptic activity and long-term potentiation (LTP) have been identified in lateral and medial perforant path^32^ and the CA1 hippocampal subfield^33^ when mice were examined at a steady state. However, another study reported that hippocampal synaptic plasticity and episodic memory were normal when assessed separately whereas CA1 LTP measured in cognitively challenged KO mice resulted stronger compared to wild-type (WT) mice^16^. Whether the CA1 LTP alteration observed in normally performing KO mice associates with dendritic spine remodeling, and if these alterations aggravate in mice exposed to tasks in which they fail to learn, is currently unknown. To bridge this information gap, we exposed KO and WT C57BL/6J mice to Novel Object Recognition (NOR) tests that were run either 1 h or 24 h after the presentation of two identical objects. Following each test, dendritic spines were measured in the lateral entorhinal cortex (LEC) and its CA1 and dentate gyrus (DG) hippocampal targets. Given the key role of LEC in novel object/context recognition^34^ and evidence that synaptic plasticity in the lateral perforant path (LPP) is altered in naive KO mice^32^, we also assessed the functional status of LEC projections to the hippocampus by comparing LPP basal synaptic activity and LTP in brain slices from NOR-exposed mice of both genotypes.

## Materials and methods

### Animals

We used 2/3-month-old male *Fmr1* KO mice on a C57BL/6J background (CAT# JAX: 003025) and wild-type C57BL/6J (CAT# JAX:000664) obtained from Charles River (Calco, Italy). *Fmr1* KO mice were bred by mating homozygous *Fmr1*^−/−^ females and hemizygous *Fmr1*^−/y^ males. Age-matched C57BL/6J mice were used as a control. All the methods and related experiments were performed in accordance with relevant guidelines and regulations (European Community Guidelines for Animal Care, Italian DL 26/2014, application of the European Communities Council Directive, 2010/63/EU, FELASA and ARRIVE guidelines), and approved by the Italian Ministry of Health and by the local Institutional Animal Care and Use Committee (IACUC). A total of 37 WT mice and 45 KO mice were used in this study.

### Novel object recognition (NOR)

The NOR test was carried out in 3-month-old KO and WT mice. Mice in their home cage were transferred to the experimental room and left to acclimate for 1 h to the new environment. NOR testing consisted of three sessions. In the first session (open field exploration), each mouse was placed in an empty squared open field (40 cm on the side) surrounded by 60 cm-high walls and left free to explore it for 10 min. The mouse was returned to its home cage for a 10-min pause during which two identical objects, i.e., glass cylinders of 3 cm in diameter and 10 cm in height, were put in opposite corners of the open field. In session 2 (training), the mouse was placed in the center of the open field and allowed to explore two similar objects (O1 and O2) for 10 min. The mouse was returned to its home cage for a 1 h-pause during which one previously explored object (familiar object: FO) was substituted with a novel object (NO), a multicolored Rubik's cube of 5 cm on the side. In session 3 (testing), the mouse was placed again in the center of the open field and allowed to explore the FO and the NO for 10 min. The NOR test was delivered either 1 h (WT mice, n = 10; KO mice, n = 14) or 24 h (WT mice, n = 13; KO mice, n = 15) after training. Mice were returned to their home cage located in the experimental room immediately after the test. Object exploration was defined as mice sniffing or touching the object with its nose and/or forepaws. All objects were cleaned with a 10% ethanol solution before their introduction in the open field. The time spent exploring each object was recorded during sessions 2 (O1 and O2) and 3 (FO and NO), and the discrimination index, the percentage of time spent exploring each object (FO or NO) divided by the total time spent exploring both objects multiplied 100, was calculated^35^. A discrimination index above 50% indicates a preference for the NO, below 50% a preference for the FO, and 50% no preference.

### Identical objects recognition (IOR)

To control that neural changes detected in KO mice following NOR actually depend on a perceived discrepancy between the training (similar objects, O1 and O2) and the test (FO vs NO), additional groups of WT (n = 4) and KO (n = 4) mice were subjected to the same three initial phases of the NOR procedure (acclimatization to the experimental room: 1 h; exploration of the empty open field: 10 min; exposure to the two similar objects—training: 10 min) but were instead re-exposed during the test to the same objects explored during training (IOR test duration: 10 min). As in the NOR procedure, mice were returned to their home cage located in the experimental room immediately after the test.

### Dendritic spines analyses

One hour and a half after being returned to their home cage, mice were deeply anesthetized with a cocktail of Zoletil (800 mg/kg) and Rompum (200 mg/kg) and perfused transcardially with 0.9% saline solution. Golgi-Cox staining was performed as previously described^36^. Spine density was measured in apical and basal dendrites of layer II LEC, dorsal CA1 pyramidal neurons, and dendrites of granule cells in the molecular layer of the DG. Neurons were identified with a light microscope (Leica DMLB) under low magnification (20×/NA 0.5) and analyzed under higher magnification (100×/NA 0.5). On each neuron, spines were counted in randomly selected five 30–100 μm dendritic segments of secondary and tertiary branch order of basal dendrites using Neurolucida software. Statistical comparisons were made on single neuron values obtained by averaging the number of spines counted on segments of the same neuron. The analysis was conducted by an experimenter blind to the experimental condition.

### Immunofluorescence assays

For immunofluorescence assays, anesthetized mice (Zoletil: 800 mg/kg/Rompum: 200 mg/kg) were perfused transcardially with 0.9% saline solution followed by the incubation of brains in 4% formaldehyde solution. Detection of presynaptic (vesicular glutamate transporter 1, vGluT1) and post-synaptic (postsynaptic density protein 95, PSD95) proteins immunoreactive puncta were carried out as previously described^37^. Primary antibodies were vesicular glutamate transporter 1 (vGlut1 Anti-Guinea Pig, 1 : 800, o/n; Synaptic System) and PSD95 (Anti-mouse, 1 : 100, o/n; Enzo Life Science). Slices (n = 3 per group) were washed three times with PBS 1× and incubated with appropriate fluorescently labeled secondary antibodies (room temperature, dark, 2 h). Slices were further washed three times in PBS 1× and then were counterstained with DAPI (1 : 1000, 10 min; Enzo Life Science). At last, sections were mounted with Fluoromount (Sigma Aldrich) and coverslipped.

### Image analysis

Signals for vGlut1 and PSD95 were detected separately from ten non-overlapping ROIs (10 × 10 µm squares) from each image; ROIs were randomly selected avoiding DAPI-labeled cell bodies. IMARIS software was used for automatic detection and counting of PSD95 and vGlut1 puncta and identification of PSD95/vGlut1 co-localization as overlapping signals. For all the experiments, ROIs were analyzed after establishing a detection threshold, which was kept constant within each measurement.

### Slice preparation and electrophysiology recordings

Mice were sacrificed by cervical dislocation, and the brains were isolated and immersed in ice-cold artificial cerebrospinal fluid (ACSF) containing (in mM): 126 NaCl, 3.5 KCl, 1.2 NaH_2_PO_4_, 1.2 MgCl_2_, 2 CaCl_2_, 25 NaHCO_3_, 11 glucose (pH 7.3) saturated with 95% O_2_ and 5% CO_2_. Parasagittal slices (400 μm) containing LEC and hippocampus were maintained at room temperature (22–24 °C) in ACSF for at least 1 h, then, each slice was transferred to a submerged recording chamber and continuously superfused at 32–33 °C with ACSF at a rate of 2.6 ml/min. Extracellular field excitatory postsynaptic potentials (fEPSPs) were recorded in the dentate gyrus with a glass microelectrode filled with 2 M NaCl solution (pipette resistance 2–5 MΩ), upon stimulation of lateral perforant path (LPP) with an insulated bipolar twisted NiCr electrode (50 μm OD). Stimulation and recording electrodes were placed in the outer part of the molecular layer of the dentate gyrus^38^, 3–4 mm apart, with the stimulation electrode at the edge of the entorhinal cortex (EC) and the hippocampal fissure. Responses were identified by application of paired-pulse stimulation (PPS, two consecutive pulses 50-ms apart), and only fEPSPs showing paired-pulse facilitation (PPF, R2/R1 ratio ≥ 1) were used for recording. Paired-pulse ratio (PPR) was used as an indicator inversely proportional to presynaptic neurotransmitter release^39^. After PPS, during normal recording, each pulse was delivered every 20 s (square pulses of 100 μs duration at a frequency of 0.05 Hz), and three consecutive responses were averaged. Signals were acquired with a DAM-80 AC differential amplifier (WPI) and analyzed with the LTP program^40^. The input/output (I/O) curves were plotted as the relationship between the fEPSP slope (mV ms^−1^) and the stimulation intensity (µA). LTP was induced by using a high-frequency stimulation (HFS) protocol consisting of two trains of 100 pulses at 100 Hz, 20 s apart. HFS was applied in disinhibited slices (10 μM bicuculline methiodide, Tocris Biosciences, Bristol, UK) to facilitate LTP expression at perforant path synapses^38^.

### Statistical analysis

As for the NOR test, the time (in sec) spent exploring each object category in sessions 2 and 3, were compared in each genotype using two-tailed Student’s t-tests for paired samples. In the IOR test, an ANOVA for repeated measures with genotype as main factor and sessions as repeated factor was used to estimate habituation of object exploration across training and test sessions. Object preference indexes were compared between genotypes using two-tailed Student’s t-tests for unpaired samples. Spine density scores and expression levels of synaptic proteins were compared using two-way ANOVAs with genotype (WT vs KO) and testing condition (NOR-tested vs naive) as main factors. Post hoc pair comparisons were carried out where necessary using the Bonferroni test. Electrophysiological data were compared using a two-tailed Mann–Whitney test for independent groups, and the Wilcoxon test for pre- vs post-bicuculline treatment comparisons. Statistical analyses and curve fittings were obtained by using Stat 3 software and GraphPad Prism software (version 6.05).

## Results

### NOR 1 h post-training

#### KO mice show NOR impairment

**Figure 1:**
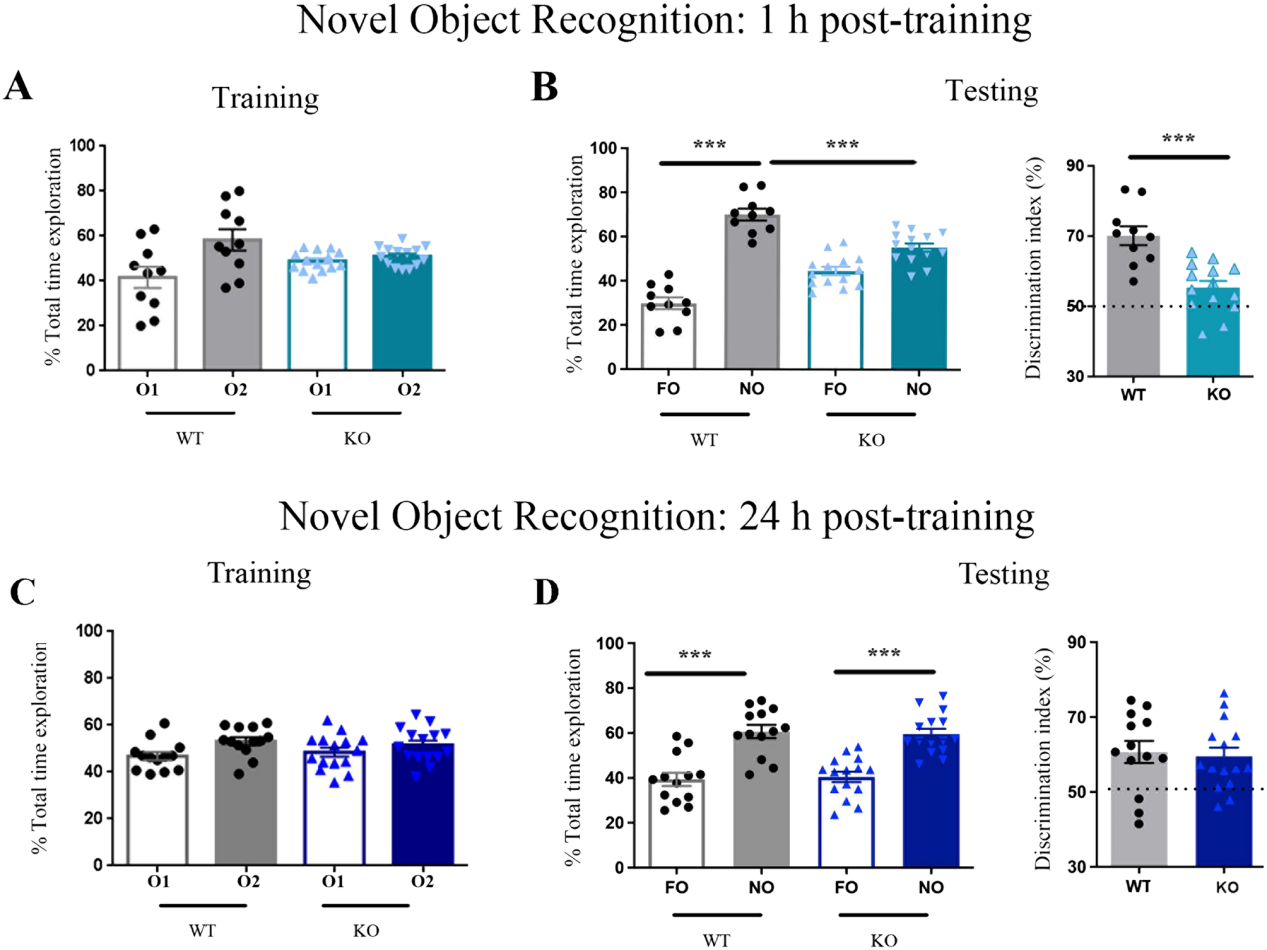
*Fmr1* KO mice show a NOR impairment at 1 h, but not 24 h, training-to-test interval. (**A**) 1 h training: Wild-type (WT: grey bars) and *Fmr1* KO (KO: blue bars) mice spend the same time exploring the identical objects (object O1, empty bars; object O2, solid bars); (**B**) 1 h testing: Left: WT mice explore the NO (grey solid bar) significantly more than the FO (grey empty bar) whereas KO mice do not show significant differences in the time spent exploring the familiar object (FO, empty light blue bar) and the novel object (NO, light blue solid bar). Right: The discrimination index, calculated as the time spent exploring the novel object divided by the total time spent exploring the two objects multiplied by 100, is significantly higher in WT mice (grey solid bar) than in KO mice (light blue solid bar). WT: n = 10; KO: n = 14. (**C**) 24 h training: Wild-type (WT: grey bars) and Fmr1 KO mice (KO: dark blue bars) spend the same time exploring the identical objects (object O1, empty bars; object O2, solid bars); (**D**) 24 h testing: Left: WT and KO mice spend more time exploring the novel object (NO, WT grey solid bar; KO, dark blue solid bar) than the familiar object (FO, WT grey empty bar; KO dark blue empty bar). Right: WT (grey solid bar) and KO (dark blue solid bar) mice exhibit a comparable object discrimination index. WT: n = 13; KO: n = 15.

KO mice exhibit learning deficits in aversively- and positively-motivated episodic memory tasks which are subtle^21^ or emerge when cognitive demand is high^23^. Compared to these protocols, non-associative spatial^22^ or object recognition^22,23^ tasks appear more appropriate to detect cognitive dysfunctions as variations in the duration of the sampling phase or the sampling-to-test interval allow to appraise the speed of sensory information processing and the recognition memory span^41^. When testing was delivered 1 h after training, we found that NOR-exposed KO (blue bar) and WT mice (grey bar) similarly explored the identical objects in session 2 (Fig. 1A, O1: empty bar vs O2: solid bar, WT: t_(20)_ = 1,6; p = 0.12; KO: t_(24)_ = 1,17; p = 0.25), whereas only KO mice failed to explore more the NO than the FO in session 3 (NO vs FO, WT t_(20)_ = 12.10, p = 0.001; KO: t_(24)_ = 2.01; p = 0.06; Fig. 1B, left panel), therefore exhibiting a significantly lower discrimination index compared to WT mice (Fig. 1B, right panel, p < 0.01).

#### IOR-exposed WT and KO mice similarly explore the identical objects during training and testing and show habituation of exploration across sessions

During training, IOR-exposed WT (grey bars) and KO (green bars) mice similarly explored the identical objects (Supplementary Fig. S1A, O1: empty bar vs O2: solid bar, WT: t_(6)_ = 0.37; p = 0.72; KO: t_(6)_ = 0.3; p = 0.23). During the test, WT and KO mice still explored similarly the identical objects (Supplementary Fig. S1B, left panel, O1 vs O2, WT t_(6)_ = 1.36, p = 0.23; KO: t_(6)_ = 0.93; p = 0.38). Accordingly, the discrimination index during the test did not differ statistically between genotypes (Supplementary Fig. S1B, right panel, WT vs KO, t_(6)_ = 1,13, p = 0.30) and was close to the 50% value which indicates no preference for any object. Remarkably, the global time of exploration significantly decreased in both genotypes between the training (WT: 30.32 s, SEM: 7.76; KO: 27.02 s, SEM: 6.66) and the test (WT: 14.42 s, SEM: 3.88; KO: 17.32 s, SEM: 3.58) sessions. Consistently with the same rate of habituation of objects exploration in both genotypes, the ANOVA revealed an effect of session (F_(1,6)_ = 7.821, p = 0.031) but no effect of genotype (F_(1,6)_ = 0.001, p = 0.97) or session × genotype interaction (F_(1,6)_ = 0.458, p = 0.52).

#### LEC spines collapse in KO mice following NOR testing

**Figure 2:**
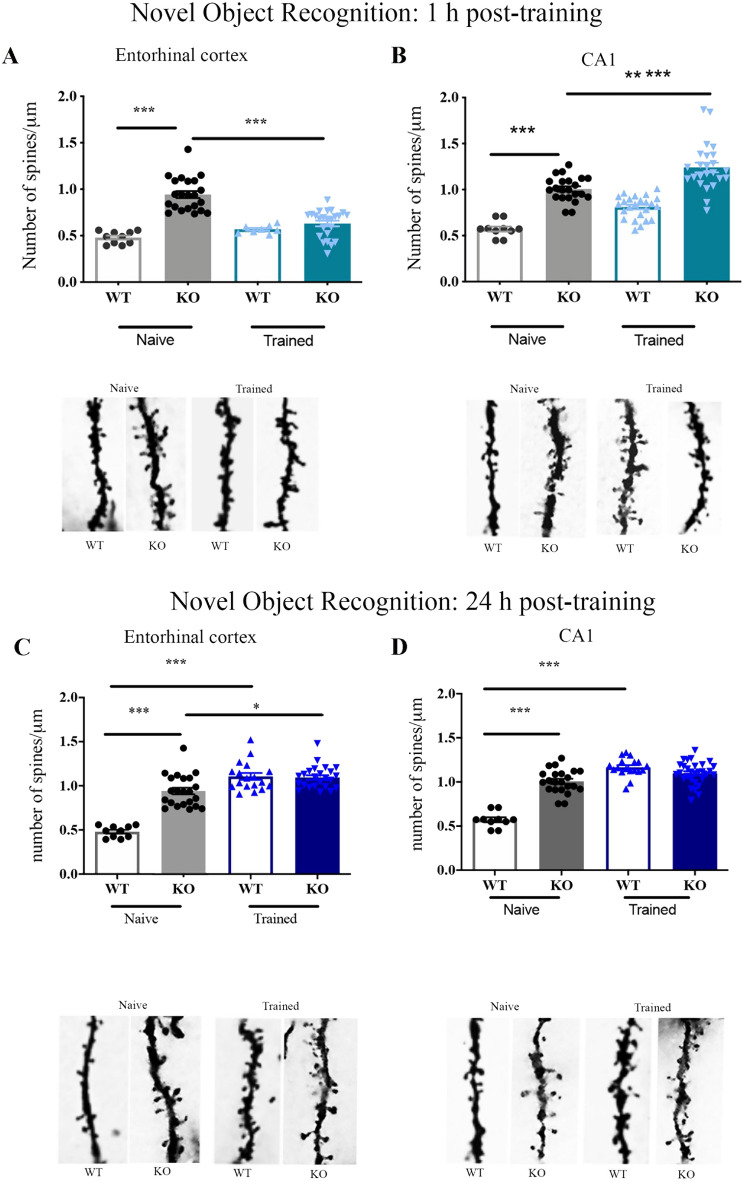
Dendritic spines and representative dendritic segments in entorhinal cortex (LEC) and CA1 pyramidal neurons from naive and NOR-tested KO mice. 1 h interval: Consistently with their immature spine morphology, naive KO mice (grey solid bars) exhibit significantly more spines than naive WT mice (grey empty bars) in both LEC (**A**) and CA1 (**B**). Following NOR failure, KO mice (light blue solid bars) show opposite remodeling in LEC and CA1 with a decrease in LEC spines and an increase in CA1 spines relative to their naive siblings (grey solid bars). Differently, in the WT mice, successful NOR does not elicit any change in spine density in any region relative to their naive siblings (NOR-tested WT mice: light blue empty bars; naive WT mice: grey empty bar). Values are expressed as the number of spines per 1 µm segment. LEC: naive WT (n = 10), naive KO (n = 22), NOR-tested WT (n = 8), NOR-tested KO (n = 22); CA1: naive WT (n = 10), naive KO (n = 22), NOR-tested WT (n = 22), NOR-tested KO (n = 24); 24 h interval: Successful NOR in KO mice was associated with a significant increase in LEC spines (**C**) but no significant change in CA1 spines (**D**) whereas in WT mice it was associated with significant increase in spines in both regions (NOR-tested KO mice: dark blue solid bars; naive KO mice: grey solid bars; NOR-tested WT mice: dark blue empty bars; naive WT mice: grey empty bars). Remarkably, at this interval where both KO and WT mice show successful NOR the spine scores of NOR-tested KO mice do not significantly differ from those of NOR-tested WT mice. LEC: naive WT (n = 10), naive KO (n = 22), NOR-tested WT (n = 22), NOR-tested KO (n = 24); CA1: naive WT (n = 10), naive KO (n = 22), NOR-tested WT (n = 24), NOR-tested KO (n = 30).

Statistical comparisons of LEC spines revealed a significant effect of genotype (F_(1,28)_ = 24,7; p < 0.001), and of the genotype × testing condition interaction (F_(1,28)_ = 13,7; p < 0.001). As shown in Fig. 2A, more LEC spines were counted in KO mice than in WT mice in the naive condition (p < 0.001). In the WT mice, no variation in LEC spines was found between NOR-exposed and naive mice (WT tested vs naive, p = 0.23) whereas a collapse of spines was found in NOR-exposed KO mice which exhibited spine scores below those of their naive counterpart (KO tested vs naive, p < 0.001).

#### CA1 and DG spines are increased in KO mice following NOR testing

CA1 spine density (Fig. 2B) was higher in KO mice compared to WT mice (significant effect of genotype, F_(1,54)_ = 19,4; p < 0.001), and in NOR-exposed compared to naive mice (significant effect of training F_(1,54)_ = 34,40; p < 0.001). Pair comparisons revealed, however, that only NOR-exposed KO mice showed a significant increase in CA1 spines compared to their respective naive counterpart (KO tested vs naive, p < 0.001; WT tested vs naive, p = 0.09). DG spines were also more abundant in naive KO mice compared to naive WT mice (p < 0.001), and in NOR-exposed KO mice compared to NOR-exposed WT mice (p < 0.05) (Supplementary Fig. S2A,C).

#### IOR testing does not affect spine density in any genotype and region

As shown in Supplementary Fig. S1C,D, no difference in spine density was detected in any region between IOR-tested and naive mice (two-way ANOVA, no effect of testing condition, LEC: F_(1,32)_ = 2.32, p = 0.14; CA1: F_(1,40)_ = 0.009, p = 0.92. In both regions, spine density was higher in KO mice than in WT mice (effect of genotype, LEC: F_(1,32)_ = 82.87, p = 0.001; CA1: F_(81.40)_ = 6.85, p = 0.012).

#### LEC and CA1 synaptic proteins are decreased in KO mice following NOR testing

**Figure 3:**
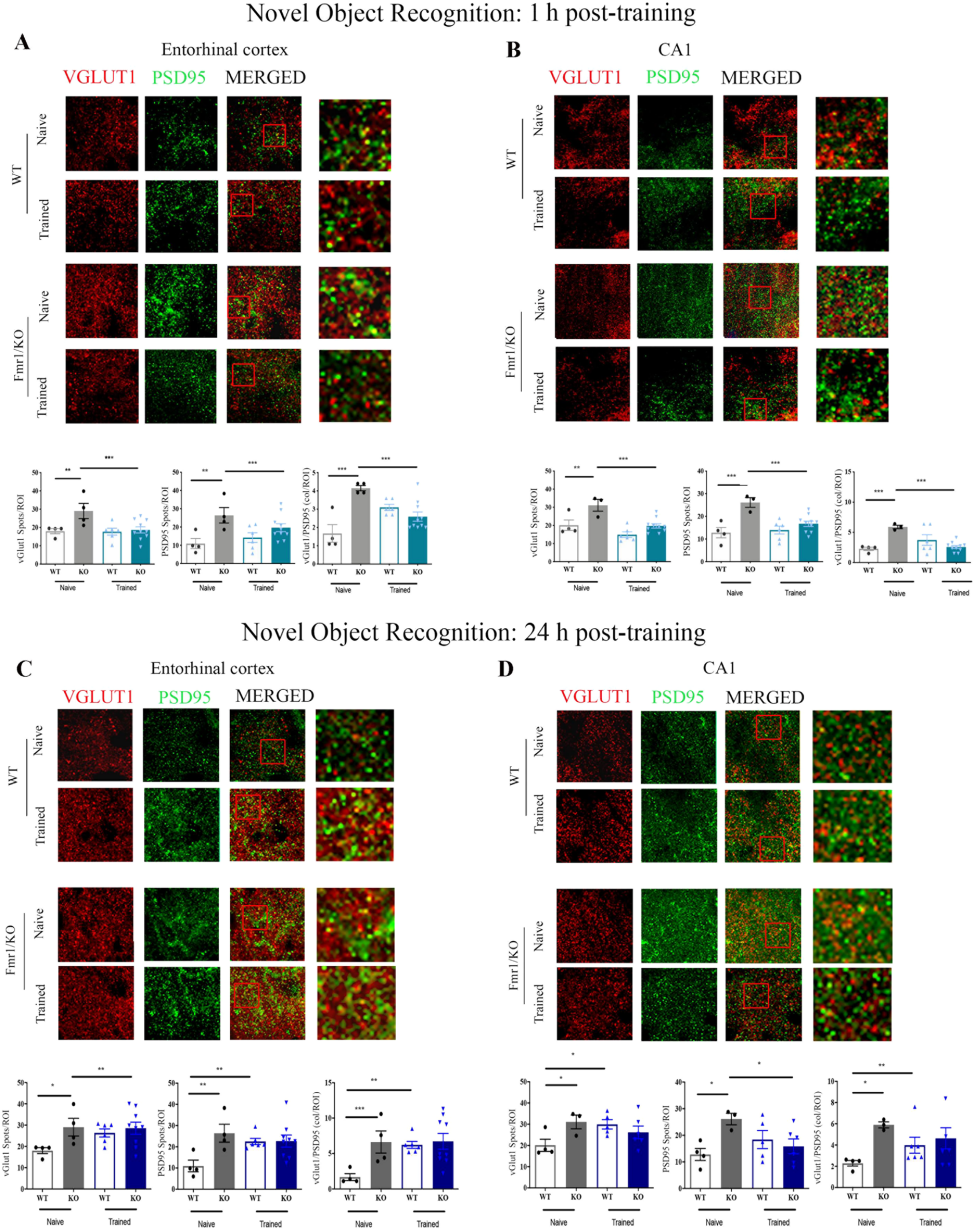
Density of immunoreactive spots and representative immunofluorescence images of presynaptic (vGluT1) and postsynaptic (PSD95) proteins in LEC and CA1 from naive and NOR-tested KO mice. (**A**,**B**) 1 h interval: Consistently with their higher spine density, naive KO mice (grey solid bars) exhibit more single-protein and co-localized immunoreactive spots than naive WT mice (grey empty bars) in both regions whereas, in line with their NOR deficit at the 1 h interval, synaptic proteins are decreased in LEC and CA1 of NOR-tested KO mice (light blue solid bars) compared to naive KO mice (grey solid bars). Differently, and in line with the absence of variations in spine density at the 1 h interval, these synaptic markers do not vary in any region between NOR-tested WT mice (blue empty bars) and naive WT mice (grey empty bars). LEC: naive WT (n = 4), naive KO (n = 4), NOR-tested WT (n = 6), NOR-tested KO (n = 10); CA1: naive WT (n = 4), naive KO (n = 3), NOR-tested WT (n = 6), NOR-tested KO (n = 10). *p < 0.05; **p < 0.01; ***p  < 0.001. 24 h interval: In both regions (**C**,**D**), naive (grey solid bars) and NOR-tested (dark blue solid bars) KO mice exhibit comparable numbers of vGluT1 and PSD95 immunoreactive spots. Differently, NOR-tested WT mice (dark blue empty bars) showed increased VGluT1 and PSD95 immunoreactivity compared to naive WT mice (grey empty bars). As for spine density measurements, the number of immunoreactive spots for each protein did not differ between NOR-tested WT mice and NOR-tested KO mice. LEC: naive WT (n = 4), naive KO (n = 4), NOR-tested WT (n = 6), NOR-tested KO (n = 9); CA1: naive WT (n = 4), naive KO (n = 3), NOR-tested WT (n = 6), NOR-tested KO (n = 7). *p < 0.05; **p < 0.01; ***p < 0.001.

ANOVAs comparing numbers of immuno-reactive puncta revealed significant genotype × NOR-exposure interactions for vGluT1, PSD95, and vGluT1/PSD95 co-localization (p < 0.01 for all comparisons) in LEC (Fig. 3A) and CA1 (Fig. 3B). In comparison with naive WT mice, naive KO mice exhibited significantly more single-protein (EC and CA1 vGluT1: p < 0.01; EC PSD95: p < 0.01, CA1 PSD95: p < 0.001) and co-localized (EC and CA1: p < 0.001) puncta. NOR-exposed KO mice exhibited a massive decrease in single-protein and co-localized puncta in LEC where we detected a collapse of spines, and, unexpectedly, in CA1 where we detected an increase in spines (p < 0.001 for all comparisons except PSD95 in LEC, p = 0.07). No variation in protein expression was found between NOR-exposed and naive WT mice.

#### LPP basal synaptic activity is normal in KO mice following NOR testing

**Figure 4:**
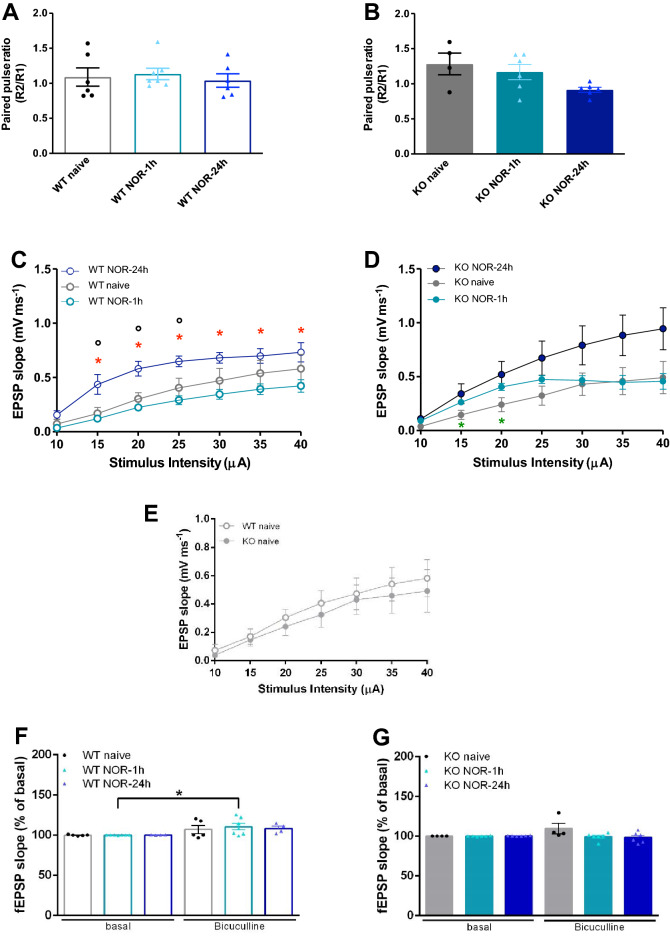
Normal PPR but higher fEPSP slopes in mice exposed to long-term NOR. (**A**,**B**) PPR measured in WT (**A**) and KO (**B**) mice are comparable regardless of the training condition (p > 0.1 for all comparisons; Mann–Whitney test). (**C**,**D**) I/O curves depict the ratio between fEPSP slope (mV ms^−1^) and the stimulation intensity (µA). Each point on the I/O curve was obtained by averaging responses over 2–5 min of recording and progressively increasing the stimulus strength. (**C**) WT mice curves are comparable in naive and NOR-1 h conditions but not in the NOR-24 h condition where the curve indicates a higher I/O ratio (°p ≤ 0.05 vs naive; *p < 0.05 vs NOR-1 h; Mann–Whitney test). (**D**) KO mice curves are also largely superimposable in naive and NOR-1 h conditions except for the 15 and 20 µA stimulation intensities which trigger higher responses in the latter group (*p < 0.05 vs naive; Mann–Whitney test). As in WT mice, the NOR-24 h curve of KO mice depicts a thoroughly higher I/O ratio compared to naive KO mice, and partially higher compared to KO NOR-1 h mice (at 30–40 µA stimulus intensities), but, in both cases, the differences were not statistically significant; (**E**) The I/O ratios in naive WT and KO mice are superimposable; (**F**,**G**) Application of bicuculline induces typical disinhibition of LPP synaptic activity in WT NOR-1 h mice (*p < 0.05, Wilcoxon test) whereas this effect is absent in WT NOR-24 h mice (**F**) and both NOR-1 h and NOR-24 h KO mice (**G**). WT naive: n = 5 slices from 3 mice; WT NOR-1 h: n = 7 slices from 3 mice; WT NOR-24 h: n = 4 slices from 3 mice; KO naive: n = 4 slices from 2 mice; KO NOR-1 h: n = 6 slices from 3 mice; KO NOR-24 h: n = 6 slices from 3 mice.

PPR did not significantly vary (p > 0.1) between NOR-exposed and naive WT mice (Fig. 4A, WT naive: 1.09 ± 0.13; WT NOR-1 h: 1.13 ± 0.08) or KO mice (Fig. 4B, KO naive: 1.28 ± 0.15; KO NOR-1 h: 1.17 ± 0.11). No variation in I/O curves was detected between naive and WT NOR-1 h mice (Fig. 4C, p > 0.1 for all comparisons), and between naive and KO NOR-1 h mice, except for a significantly higher averaged fEPSP slope found in KO NOR-1 h mice at 15 and 20 µA stimulus intensities (Fig. 4D, p < 0.05). I/O curves of WT and KO naive mice were also superimposed (Fig. 4E).

#### Bicuculline-induced disinhibition of LPP synaptic activity is absent in KO mice following NOR testing

Consistently with data showing that application of the γ-Aminobutyric acid type A (GABAA) receptor antagonist bicuculline methiodide (10 µM) potentiates fEPSP and facilitates induction of synaptic plasticity at perforant path synapses^39^, we observed a significant increase in LPP synaptic activity in bicuculline methiodide-treated slices from WT NOR-1 h mice (Fig. 4F, 110.4 ± 3.9% of basal slope, p < 0.05 vs pretreatment basal level) that was not present in KO NOR-1 h mice (Fig. 4G, 99.4 ± 1.9% of basal slope, p > 0.1 vs pretreatment basal level) consistently with the reduced GABAergic inhibitory tone in their hippocampus^42–44^.

#### LPP-LTP collapses in KO mice following NOR testing

**Figure 5:**
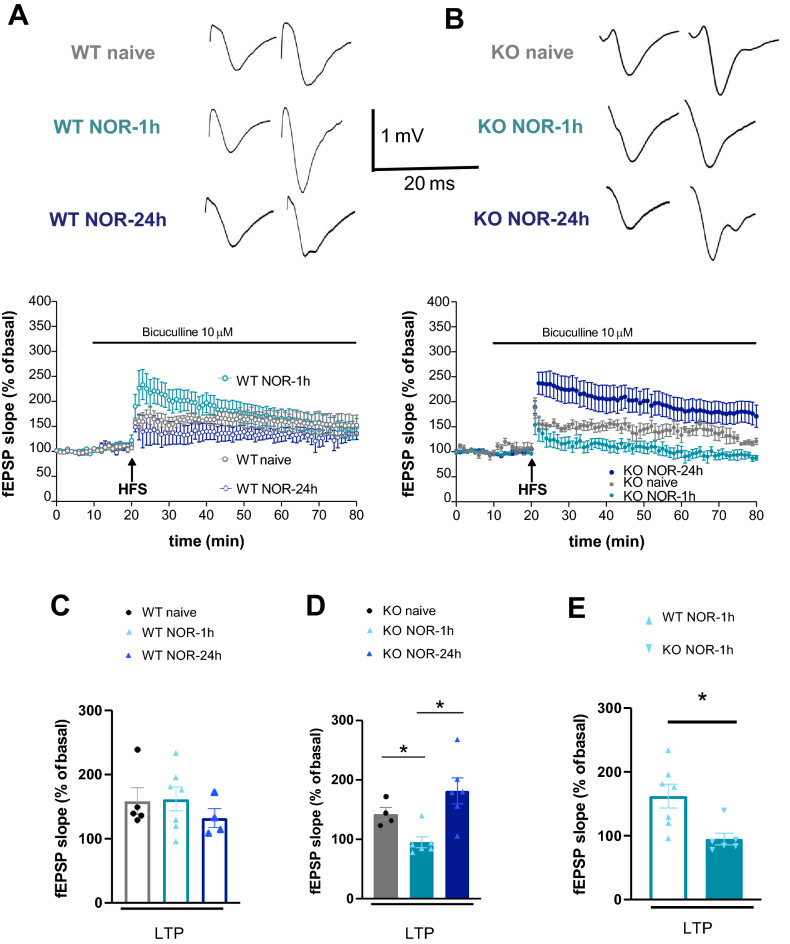
LPP-LTP is occluded in KO mice exposed to NOR-1 h but abnormally elevated in KO mice exposed to NOR-24 h. Data were expressed as mean ± SEM of n experiments (one slice tested per experiment). Slices were obtained from at least two mice for each experimental set. Slope values were normalized, taking the average of the baseline values as 100%. The LTP induction and magnitude were expressed as the mean percentage variation of the slope from baseline calculated in the time windows 0–20 and 40–50 min after HFS, respectively. Synaptic potentiation in WT (**A**) and KO (**B**) mice was measured in different conditions. Upper insets in (**A**) and (**B**) panels show representative fEPSP recordings in the baseline condition (left) and 40 min after LTP induction (right). Calibration bars: 1 mV, 20 ms. (**A**) WT mice show similar potentiation regardless of the training condition. (**B**) In the KO mice, an LTP decrement is found in the NOR-1 h condition (p < 0.05 vs naive, light blue vs grey solid dots; Mann–Whitney test) whereas an exaggerated LTP is found in the NOR-24 h condition (p < 0.01 vs naive, dark blue vs grey solid dots; Mann–Whitney test). (**C–E**) The graphs summarize the LPP-LTP results.

In WT mice, LTP magnitude was comparable in each group regardless of exposure or non-exposure to NOR (Figs. 5A,C, WT naive: 158.1 ± 20.5% of basal slope; WT NOR-1 h: 161.5 ± 18.3% of basal slope, p > 0.1). Differences were instead found in NOR-exposed KO mice, with an LTP decrement being observed in KO NOR-1 h mice compared to KO naive mice (Fig. 5B,D, KO naive: 142.2 ± 10.7% of basal slope; KO NOR-1 h: 95.03 ± 9.2% of basal slope, p < 0.05), and to WT NOR-1 h mice (Fig. 5E). Similar findings were found for LTP induction (Supplementary Fig. S3).

### NOR 24 h post-training

#### NOR performance does not vary between genotypes

WT (grey bar) and KO (dark blue bar) mice similarly explored the similar objects during the training phase (Fig. 1C, O1 (empty bar) vs O2 (solid bar), WT: t_(20)_ = 1,5; p = 0.15; KO t_(24)_ = 1,17; p = 0.25), and explored more the NO than the FO during the testing phase (Fig. 1D, NO vs FO, WT: t_(20)_ = 4.2, p = 0.001; KO: t_(24)_ = 6.99; p < 0.001), with both strains exhibiting a comparable discrimination index (WT vs KO, p > 1).

#### LEC, CA1, and DG spines do not vary between genotypes following NOR testing

For the LEC spines (Fig. 2C), there was a significant effect of genotype (F_(1,63)_ = 11,97; p < 0.001), NOR exposure (F_(1,63)_ = 46,54; p < 0.01), and of the genotype × NOR exposure interaction (F_(1,63)_ = 13,87; p < 0.001). LEC spines were still more numerous in KO naive than in WT naive mice (p < 0.001) but, in contrast with the NOR 1 h data, EC spines were increased post-training in both genotypes (WT tested vs naive, p < 0.01; KO tested vs naive, p < 0.05). Remarkably, the spine scores of NOR-exposed WT and KO mice were in the same range (WT tested vs KO tested, p > 1). For the CA1 spines (Fig. 2D) there was also a significant effect of genotype (F_(1,66)_ = 30.88; p < 0.001), NOR-exposure (F_(1,66)_ = 84,60; p < 0.001), and of the genotype x NOR exposure interaction (F_(1,66)_ = 56,01; p < 0.001). Naive KO mice exhibited more CA1 spines than naive WT mice (p < 0.01). NOR exposure increased CA1 spines in WT mice (WT tested vs WT naive, p < 0.001) but did not in KO mice (KO tested vs KO naive, p = 0.23). Nevertheless, as for the LEC spines, CA1 spines did not vary between WT tested and KO tested mice (p > 1). Similar findings were found for spines counted in the DG (Supplementary Fig. S2B,C).

#### LEC and CA1 synaptic proteins do not vary between genotypes following NOR testing

The same pattern of synaptic proteins expression was observed in LEC (Fig. 3C) and CA1 (Fig. 3D). There was a significant genotype × NOR exposure interaction for vGluT1, PSD95, and VGluT1/PSD95 co-localization in LEC and CA1 (p < 0.05 for all comparisons). In each region, the level of each protein and their co-localization were higher in naive KO mice than in naive WT mice (p < 0.01 for all comparisons). These levels were further increased in WT tested mice compared to naive WT mice (LEC: vGluT1, p < 0.05; PSD95 and vGluT1/PSD95, p < 0.01; CA1: vGluT1, PSD95 and vGluT1/PSD95, p < 0.05 for all comparisons). Differently, NOR-exposed KO mice did not show any increase in synaptic proteins since their levels were in the same range as those of NOR-exposed WT mice (p > 1 for all comparisons).

#### KO mice show normal PPR but higher fEPSP slopes following NOR testing

NOR 24 h post-training did not affect PPR in WT mice (Fig. 4A, WT naive: 1.09 ± 0.13; WT NOR-24 h: 1.04 ± 0.1; p > 0.1) nor in KO mice (Fig. 4B, KO naive: 1.28 ± 0.15; KO NOR-24 h: 0.91 ± 0.04; p > 0.1), although a trend toward a diminution in trained vs naive KO mice (Fig. 4B, p > 0.1) suggests some increase in the presynaptic contribution to early LTP. I/O plots revealed higher averaged fEPSP slopes both in WT NOR-24 h mice (Fig. 4C) and KO NOR-24 h mice (Fig. 4D) compared to their naive counterparts but this difference was significant only in the WT NOR-24 h mice (p ≤ 0.05 vs WT naive; p < 0.05 vs WT NOR-1 h). Of note, no bicuculline-induced enhancement of synaptic activity was detected in the trained mice of both genotypes (Fig. 4F, WT NOR-24 h: 108.1 ± 2.9% of basal slope, p > 0.1 vs pretreatment basal level; Fig. 4G, KO NOR-24 h: 98.2 ± 2.7% of basal slope, p > 0.1 vs pretreatment basal level).

#### KO mice show exaggerated LPP-LTP following NOR testing

LTP magnitude did not differ between naive and trained WT mice (Fig. 5A,C, WT naive: 158.1 ± 20.5% of basal slope; WT NOR-24 h: 131.9 ± 14.4% of basal slope; p > 0.1) and between naive and trained KO mice (Fig. 5B,D, KO naive, 142.2 ± 10.7% of basal slope; KO NOR-24 h, 181.5 ± 21.8% of basal slope; p > 0.1), but was significantly higher in KO NOR-24 h than in KO NOR-1 h mice (Fig. 5B,D, KO NOR-24 h, 181.5 ± 21.8% of basal slope; KO NOR-1 h: 95.03 ± 9.2% of basal slope; p < 0.01). Comparable results were found for LTP induction and are reported in Supplementary Fig. S3.

## Discussion

Consistent with the view that memory impairments in FXS^20–23^ or other mouse models of intellectual disabilities^45^ need more time to learn, we found that KO mice fail to detect object novelty when the training-to-test interval is fixed at 1 h whereas they succeed when the interval is extended to 24 h. It is therefore apparent that augmenting the time which separates the presentation of similar and different objects does not render the memory of the familiar object weaker but instead facilitates the initial encoding of object similarity as well as subsequent detection of object novelty. Confirming that NOR failure depends on a selective deficit in the perception of a discrepancy between the presentation of similar (training) and different (testing) objects, control experiments in which KO and WT mice were exposed to the same configuration of identical objects during training and testing revealed no genotype difference in the rate of object exploration in any session and robust and similar habituation of objects exploration across sessions.

Successful NOR has been shown to depend on close interactions between neurons in perirhinal/lateral entorhinal cortices, which are object detectors^46^, and hippocampal neurons, which compare and identify mismatches between current information vs previously formed environmental representations stored in parahippocampal/neocortical regions^47,48^. By showing that NOR failure is associated with utmost disruption of plasticity upstream the hippocampus and that LEC spines and LPP-LTP plasticity are not merely refractory to experience-dependent changes but collapse when mice are tested 1 h after training, our data reveal that KO mice have a primary impediment in elaborating and encoding representations of similar/different object characteristics. The origin of this impediment likely stands in the defective processing of sensory information extensively reported in FXS individuals and mouse models^49^ which has been ascribed to hyperexcitable cells and circuits^50,51^ and immature synapses^52^ in primary sensory regions. Of note, the similar habituation of object exploration observed in KO and WT mice exposed to the IOR test did not elicit changes in LEC or CA1 spines in any genotype. Specifically, spines did not vary between IOR-tested KO and WT mice although they were still more abundant in KO mice than in WT mice.

Assuming that a distorted sensory input reaches temporal lobe regions, surprisingly, we found that it triggers differential structural remodeling of entorhinal and hippocampal synapses. The paradoxical aspect of differential spine remodeling in LEC and CA1 is that these neurons show the same baseline morphological abnormalities-abundant immature spines, and exhibit the same form of reactive structural plasticity when exposed to enriched environment stimulation^7,9,53^. Confirming that region-specific differential remodeling is actually triggered by NOR failure, repeated exposure to similar objects during training and testing did not elicit changes in LEC or CA1 spines in any genotype.

Differently, WT and KO mice achieve successfully NOR when a 24 h interval is interposed between training and testing, and exhibit similar spine scores in LEC and CA1. This similarity depends, however, on the fact that exposure to Long-Term NOR brings the density of WT spines to the level constitutively expressed by KO mice. Thus, no elevation of spine density imputable to successful performance is detected in this genotype, consistently with the report that disease-associated spine changes above (autism spectrum disorders) or below (Alzheimer's disease) a physiological threshold alter the functional state neural circuits and the cognitive operations they support^54^. Remarkably, there was a good alignment between variations, or no variation, in spines and synaptic protein levels in this NOR condition. Specifically, NOR-tested WT mice showed increased levels of vGluT1, PSD95, and their co-localization in LEC and CA1, consistently with the augmentation of spines in these regions, whereas NOR-tested KO mice did not show variations in synaptic proteins, consistently with the stability of spines in these regions. Minor deviations from this alignment like the only increase in vGluT1 in the LEC of WT mice, or the decrease in PSD95 in the CA1 of KO mice do not invalidate this central observation. Interestingly, exaggerated induction and magnitude of LPP-LTP, which is observed in the unique condition where KO mice show an increase in LEC spines, remain a peculiarity of NOR exposed KO mice. This phenomenon can be viewed as some form of neural compensation^55^ since, because of the immature spines and synaptic protein defects observed in the region, the LPP-LTP overshooting is likely required to drive a sufficient amount of structural remodeling warranting successful NOR.

In the continuous flow of sensory stimuli that every living organism perceives in its environment, the ability to distinguish between new and familiar stimuli is of crucial adaptive importance. Computational^56^ and physiological^57,58^ models of the mammalian brain posit that the hippocampus is central to novelty detection because its anatomical circuitry is ideally featured to detect mismatches between incoming sensory inputs and previously encoded representations. The prerequisite of these models is that the information which is detected and stored within perirhinal and entorhinal cortices during object exploration is regularly conveyed to the hippocampus by the perforant path. The point is that perirhinal and entorhinal cortices are not simple nodes where sensory information transits before it reaches the hippocampus, but regions where representations of objects are formed and temporarily stored^59^. Thus, the collapse of entorhinal synapses and LPP are expected to mimic the effect produced by disconnecting the entorhinal cortex from the hippocampus^60^ to the extent that little specific sensory information regarding objects reaches, and is processed by, the hippocampus. This jeopardizes the function of constitutively fragile, but structurally plastic, nodes downstream of the LEC like CA1 and DG, which fail to support meaningful representations^61^ due to the insufficiency of incoming explicit sensory information.

A limitation of the present study might be that a behaviorally induced modulation of neuroplasticity should be confirmed by showing causal inverse relationship between neuroplasticity and behavior. Nevertheless, given the opposite modifications of plasticity detected in LEC vs CA1, attempts to correct the behavioral impairment in KO mice or to generate it in WT mice would require to concurrently perform region-specific opposite (long-term depression and LTP) manipulations whose effects on behavior might be difficult to control and interpret.

As previously emphasized^62^, NOR studies in rodent models of cognitive disabilities have analyzed considerably more memory than perception, affordances, and formation of object representations. Because basic sensory and memory circuits are conserved across mammalian species^49^, we believe that preclinical studies showing how atypical/distorted sensory inputs are processed by entorhinal-hippocampal regions in FXS mice models can shed light on neural mechanisms at the origin of intellectual disabilities in FXS individuals.

In particular, we observed that a short interval inserted between the presentation of similar and dissimilar objects, that does not allow mice to perceive the gap between the two object configurations, is associated with a post-test collapse of LEC synapses. This demonstrates that hemizygous deletion of the *Fmr1* gene affects the processing of sensory information more severely than the associative operations, the comparison, and storage of object representations, depend on. Acknowledging the limited translational potential of the present findings, far from the understanding of the neural mechanisms behind learning impairment or improvement in FXS, our results however suggest that rehabilitation strategies combining temporal lobe cortical stimulation with the presentation of learning tasks that systematically facilitate the discrimination and encoding of sensory stimuli might be beneficial for FXS individuals.

## Supplementary Information

Supplementary Information.

## Data Availability

The data will be made available from the corresponding authors (Alberto Martire and Martine Ammassari-Teule) upon acceptance for publication on reasonable request.
