# Supplementary material for: Fmr1-KO mice failure to detect object novelty associates with a post-test decrease of structural and synaptic plasticity upstream of the hippocampus
Source: Sci Rep. 2023 Jan 14;13:755. doi: 10.1038/s41598-023-27991-9 (PMC9840621; doi:10.1038/s41598-023-27991-9)

*Scientific Reports – Supplementary Information*

***Borreca et al.***

## Supplementary Figures and Legends

**Supplementary Figure S1.** WT and KO mice show the same rate of object exploration during training and testing, the same decrease of object exploration between training and testing, and the same spine scores as their respective naive controls. (**A**) During IOR training, wild-type (WT: grey bars) and *Fmr1* KO (KO: blue bars) mice spent the same time exploring the identical objects (object O1, empty bars; object O2, solid bars). **(B)** *Left*: During IOR testing, WT and KO mice spent the same time exploring the two identical/familiar objects (WT: O1 and O2, grey striped bars; KO: O1 and O2: blue striped bars). *Right*: The discrimination index, calculated as the time spent exploring each familiar divided by the total time spent exploring the two objects multiplied by 100, does not significantly differ between WT mice (grey striped bar) and KO mice (blue striped bar). **(C-D)** Spine density scores and representative images of dendritic spines were measured and taken in LEC **(C)** and CA1 **(D)** pyramidal neurons from naive and IOR-tested WT and KO mice. In each region, IOR-tested WT and KO mice (blue striped bars) did not exhibit changes in spine compared to their naive control groups (grey striped bars). Spine density was significantly higher in KO mice than in WT mice regardless of the testing condition. Values are expressed as the number of spines per 1 μm segment. LEC and CA1: naive WT (n = 3 mice, 7 neurons in LEC and CA1), naive KO (n = 3 mice, 8 neurons in LEC and 10 neurons in CA1), IOR-tested WT (n = 4 mice, 9 neurons in LEC and 12 in CA1), IOR-tested KO (n = 4 mice, 12 neurons in LEC and 16 neurons in CA1). Post hoc pair comparisons * $p < 0.05$, *** $p < 0.001$.

**Supplementary Figure S2.** Dentate gyrus (DG) spines are more numerous in KO mice than in WT mice both in the naive and the NOR-1h condition. Data from naive WT and KO mice are reported both in **A** and **B**. Spine density scores (dot-plot graphs) measured in WT and KO mice following NOR 1h (**A**) and 24h (**B**) post-training. Representative images at low (20 ×/NA 0.5) and higher (100 ×/NA 0.5) magnification of spines in DG granule cells dendrites from naive and NOR-tested WT and

KO mice (**C**). In the naive condition, KO mice (grey solid bars) showed more spines than WT mice (grey open bars). In the NOR-1h condition, NOR-tested KO mice (blue solid bars) show more spines than NOR-tested WT mice (blue open bars). In the NOR-24h condition, both NOR-tested WT mice (dark blue open bars) and KO mice (dark blue solid bars) show the same spine scores. * $p < 0.05$; *** $p < 0.001$. Values are expressed as the number of spines per 1 μm segment. WT naive (n= 10), KO naive (n = 21), WT NOR-1h (n = 20), KO NOR-1h (n = 24), WT NOR-24h (n = 18), KO NOR-24h (n = 30). Two-tailed Student's t-tests for unpaired samples.

**Supplementary Figure S3.** LTP induction data confirm that synaptic plasticity in KO mice is decreased in NOR-1h condition, and enhanced in NOR-24h condition**. (A)** WT mice data show that LTP induction is comparable in the naive condition (grey open bar) vs NOR-1h (blue open bar) and NOR-24h (dark blue open bar) conditions. A comparison of NOR-tested groups reveals, however, a significant decrease in the NOR-24h vs NOR-1h condition; **(B)** KO mice data show that LTP induction is significantly higher in the NOR-24h condition (dark blue solid bar) than in the NOR-1h (blue solid bar, $p < 0.01$) and the naive (grey solid bar, $p < 0.05$) conditions (Mann-Whitney test). Genotype comparisons in each NOR condition reveal that (**C**) LTP induction is lower in KO than in WT mice tested for NOR-1h and (**D**) higher in KO than in WT mice tested for NOR-24h. * $p < 0.05$, Mann-Whitney test. A more detailed description of LTP induction results is reported below:

*NOR-1h*

LTP induction was comparable in each genotype regardless of training (Supplemental Figure S3A, WT naive: 162,1 ± 10,3% of basal slope, n = 5 slices from 3 mice; WT NOR-1h: 207,2 ± 23,3% of basal slope, n = 7 slices from 3 mice, $p > 0.1$; Supplemental Figure S3B, KO naive: 153,2 ± 4,8% of basal slope, n = 4 slices from 2 mice; KO NOR-1h 121,3 ± 13,1% of basal slope, n = 6 slices from 3 mice; $p > 0.1$). The comparison of LTP induction between WT and KO-trained mice revealed a significant decrement in the latter group (Supplemental Figure S3C, WT NOR-1h 207,2 ± 23,3% of the basal slope; KO NOR-1h: 121,3 ± 13,1% of basal slope, $p < 0.01$).

*NOR-24h*

In WT mice, LTP induction was comparable in naive and WT NOR-24h mice (Supplemental Figure S3A, WT naive: 162,1 ± 10,3% of basal slope, n = 5 slices from 3 mice; WT NOR-24h: 138,8 ± 21,2%

of basal slope, n = 4 slices from 3 mice, $p > 0.1$), while it was significantly reduced in WT NOR-24h mice in comparison to WT NOR-1h mice ($p < 0.05$). As shown in Supplemental Figure S3B, LTP induction resulted significantly higher in trained KO mice than in both naive KO mice (KO NOR-24h: 219,8 ± 24,8% of basal slope, n = 6 slices from 3 mice; KO naive: 153,2 ± 4,8% of basal slope, n= 4 from 2 mice, $p < 0.05$) and KO NOR-1h mice (KO NOR-1h: 121,3 ± 13,1% of basal slope vs KO NOR-24h, $p < 0.05$). Of note, a comparison of NOR-1h and NOR-24h data reveals that massed and distributed training triggered opposite effects on LTP induction in the KO mice. NOR-1h training decreased LTP induction (Figure S3C, KO NOR-1h vs WT NOR-1h, $p < 0.05$) whereas NOR-24h training increased it (Figure S3D, KO NOR-24h vs WT NOR-24h, $p < 0.05$).

Supplementary Figure S1

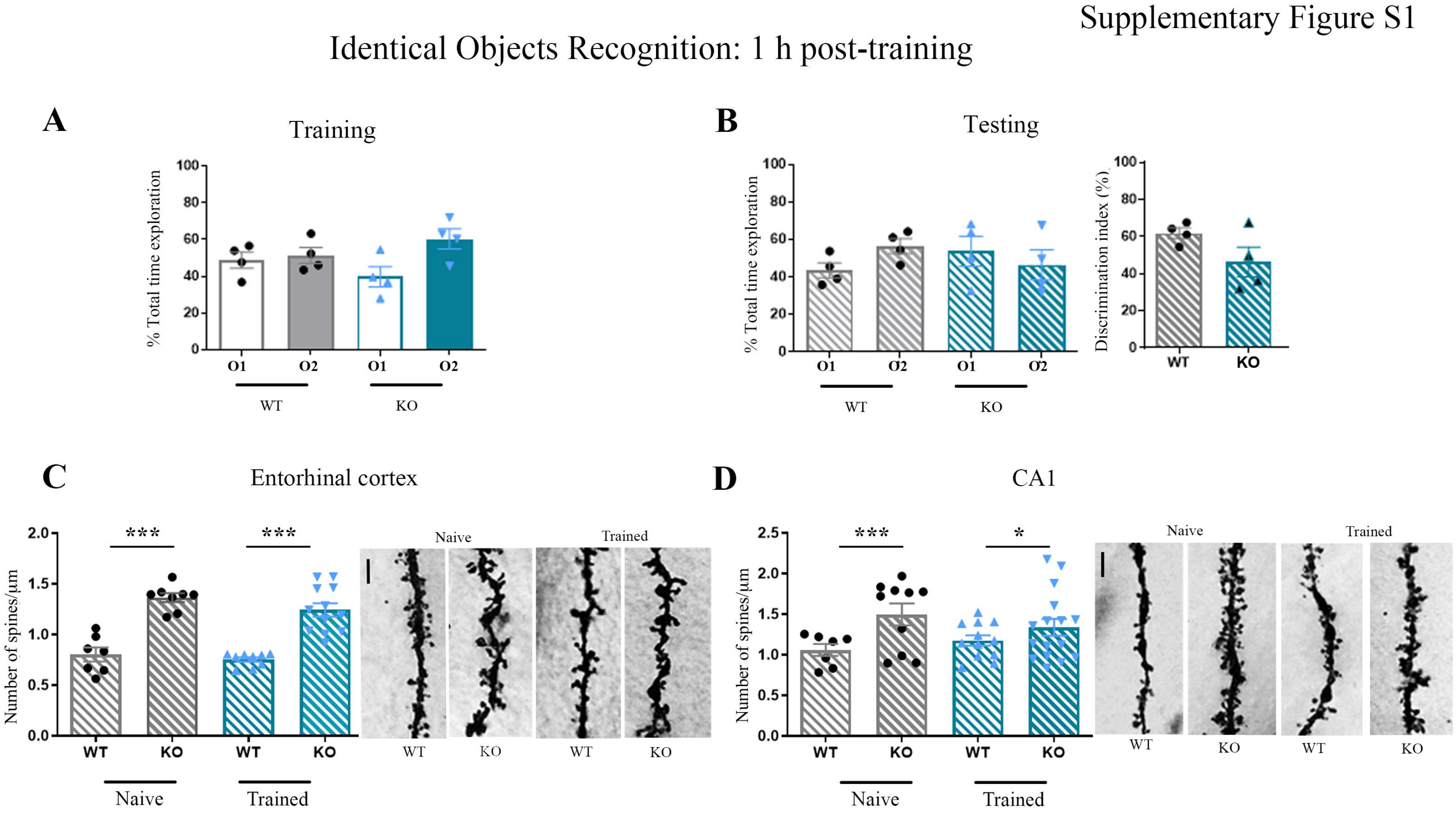

Supplementary Figure S2

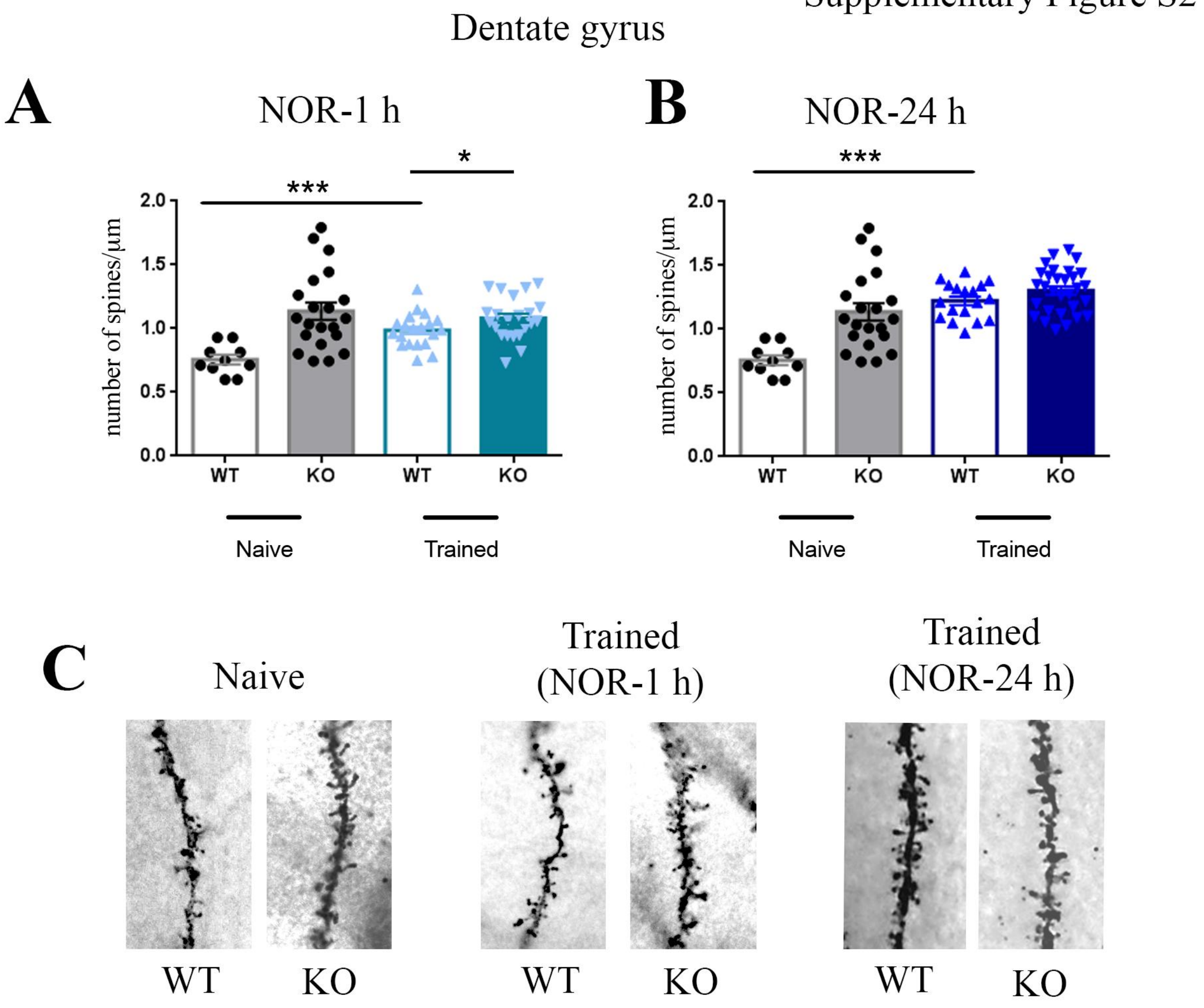

# Supplementary Figure 3

## LTP induction

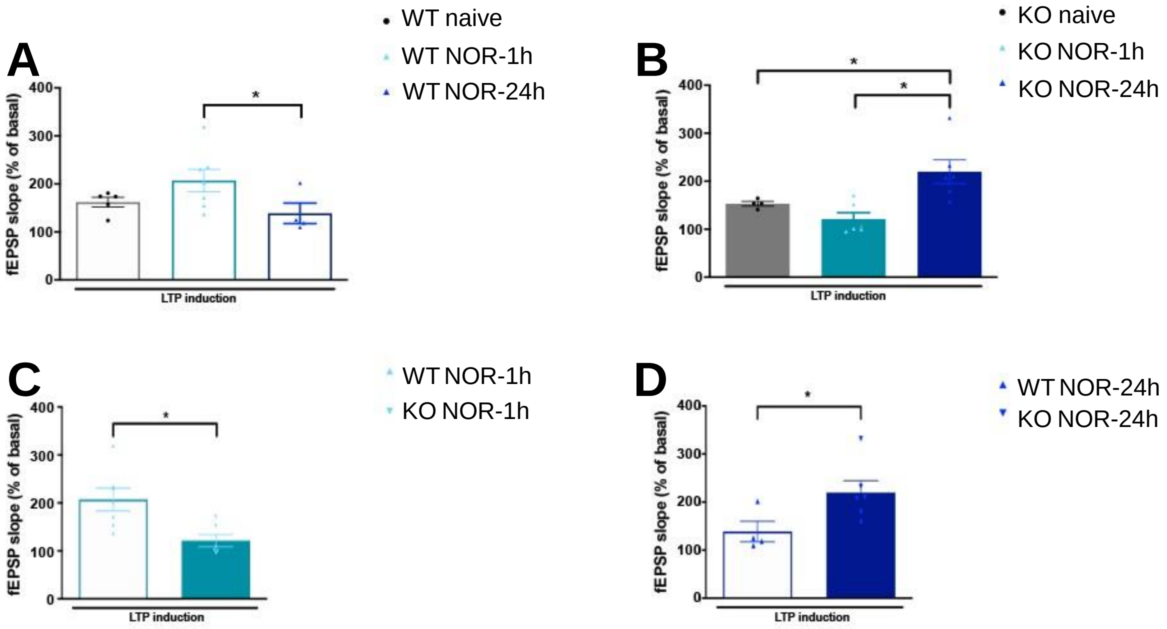

Supplement: Supplementary file 1 — Supplementary Information. [file 41598_2023_27991_MOESM1_ESM.pdf]
